# Understanding the role of Aβ and Tau in neuronal vulnerability in models of sporadic Alzheimer's disease

**DOI:** 10.1002/alz70855_100973

**Published:** 2025-12-23

**Authors:** Féodora Bertherat, Becky C. Carlyle, Nora Bengoa‐Vergniory, Sally A Cowley, Richard Wade‐Martins

**Affiliations:** ^1^ University of Oxford, Oxford, United Kingdom; ^2^ University of Oxford, Oxford, Oxfordshire, United Kingdom; ^3^ Achucarro Basque Center for Neuroscience, Leioa, Basque Country, Spain; ^4^ James and Lillian Martin Centre for Stem Cell Research, University of Oxford, Oxford, United Kingdom

## Abstract

**Background:**

This project investigates whether cellular phenotypes observed in iPSC‐derived cortical neurons of individual sAD patients can be compared with the clinical measures of the patient they originate from. It also aims to investigate resilience and vulnerability to Amyloid Beta by examining transcriptomic differences between patient lines after AB insult.

**Method:**

This project uses iPSC lines derived 14 sporadic Alzheimer's disease patients for which we have many clinical measures, including PET, MRI scans, cognitive tests, and CSF. The iPSCs lines were differentiated into cortical neurons using a doxycycline‐inducible Ngn2 system. They were treated with AB oligomers. We also performed bulk RNA‐sequencing of these cortical neurons and performed downstream analyses in RStudio.

**Result:**

Neurons from the 14 sAD patientswere transduced with an NgN2‐GFP lentivirus, were live‐imaged for 7 days post AB insult to assess neurite loss. While some patient line exhibit 80% neurite loss and others remain largely unaffected, with 10‐12% loss. This spectrum of vulnerability correlates with clinical measures of the patients, such as hippocampal volume.

We performed RNA‐sequencing of iPSC‐derived i3 cortical neurons, to establish whether AB insult would lead to transcriptional perturbations of the cortical neurons. AB oligomer treatment robustly perturbs lipid synthesis and metabolism pathways, as well as synapse formation, neuronal migration and proteasomal pathways.

Given these results, we recently performed bulk RNA‐sequencing of 14 sAD lines treated with AB oligomers, with the goal of identifying differentially expressed genes or pathways that explain the range of vulnerability observed in the sAD patient lines. The results from this latest study will be presented at this conference.

**Conclusion:**

iPSC lines respond differently to Amyloid b (Ab) insult, and this inter‐individual variation in vulnerability in cellular phenotypes such as neurite outgrowth correlates with clinical measures such as hippocampal volume.

AB induces robust transcriptomic changes of i3 cortical neurons, including lipid metabolism and proteosomal perturbations and potential changes in gene expression due to AB insult in the sAD patients neurons is being further investigated.

